# Using 100 Hz Mastoid Vibration in Apogeotropic Posterior Semicircular Canal Benign Paroxysmal Positional Vertigo: Diagnostic and Therapeutic Implications in a Retrospective Case–Control Study

**DOI:** 10.3390/audiolres16040112

**Published:** 2026-08-03

**Authors:** Giuseppe Santopietro, Luigi Califano, Iacopo Cangiano, Cataldo Latorre, Maria Grazia Melillo, Roberto Teggi

**Affiliations:** 1 Audiology and Phoniatrics Department, AORN San Pio, Via dell’Angelo 1, 82100 Benevento, Italy; giuseppe.santopietro312@gmail.com (G.S.); grazia601@live.com (M.G.M.); 2 Department of Otolaryngology, IRCCS San Raffaele Scientific Institute, San Raffaele Hospital, Via Olgettina 60, 20132 Milan, Italy; cangiano.iacopo@hsr.it (I.C.); aldo.latorre@gmail.com (C.L.); teggi.roberto@hsr.it (R.T.)

**Keywords:** benign paroxysmal positional vertigo, posterior semicircular canal, apogeotropic variant, mastoid vibration, skull-vibration-induced nystagmus test, repositioning maneuvers

## Abstract

**Background**: Positional torsional downbeating nystagmus elicited during diagnostic maneuvers for posterior semicircular canal benign paroxysmal positional vertigo (PSC-BPPV) may indicate either ipsilateral apogeotropic PSC-BPPV or contralateral anterior canal BPPV, making differential diagnosis challenging. This retrospective study evaluated whether ipsilateral 100 Hz mastoid vibration could facilitate conversion to a geotropic nystagmus pattern, thereby supporting both diagnosis and treatment. **Materials and Methods**: Ten patients with apogeotropic PSC-BPPV underwent standard diagnostic and therapeutic maneuvers combined with ipsilateral 100 Hz mastoid vibration. The primary outcome was the rate of nystagmus geotropization. Secondary outcomes included the number of repositioning maneuvers required to achieve complete symptom resolution. Outcomes were compared with those of two control groups: one consisting of patients with apogeotropic PSC-BPPV treated without mastoid vibration and another consisting of patients with typical PSC-BPPV treated using standard repositioning maneuvers alone. **Results**: Mastoid vibration induced geotropization in 7 out of 10 patients (70%). The mean number of liberatory maneuvers required for symptom resolution was lower in vibration-responsive patients than in vibration-non-responsive patients (1.7 ± 0.49 vs. 3.0 ± 1.41;). Compared with patients with apogeotropic PSC-BPPV treated without mastoid vibration, vibration-responsive patients required significantly fewer maneuvers (2.1 ± 0.87 vs. 2.9 ± 0.74; *p* = 0.036). No significant difference was observed when compared with patients with typical PSC-BPPV (2.1 ± 0.87 vs. 2.1 ± 0.52; *p* = 0.82). **Conclusions:** Mastoid vibration may represent a useful adjunctive tool in the management of apogeotropic PSC-BPPV by facilitating conversion to the typical geotropic pattern. This conversion may provide useful diagnostic information, and potentially reduce the number of repositioning maneuvers required to achieve symptom resolution.

## 1. Introduction

Positional torsional downbeating nystagmus elicited during the Dix–Hallpike maneuver may indicate either ipsilateral apogeotropic posterior semicircular canal benign paroxysmal positional vertigo (PSC-BPPV) or contralateral anterior canal BPPV [1,2,3]. This diagnostic overlap frequently complicates differential diagnosis during routine vestibular assessment, particularly in atypical clinical presentations.

Several studies have identified subtle clinical differences between these two conditions. In anterior canal BPPV, the vertical downbeating component may predominate, with minimal or absent torsional features, whereas in apogeotropic PSC-BPPV the torsional component is typically more pronounced and directed toward the uppermost ear [1,2,3]. However, these characteristics are not sufficiently reliable to establish a definitive diagnosis in all cases. Consequently, failure of liberatory maneuvers, such as the Demi-Semont maneuver, should prompt consideration of alternative diagnoses, including either anterior canal involvement or central diseases. Additional diagnostic tools, such as vertical canal video head impulse testing (vHIT), may improve diagnostic accuracy in selected cases [4].

The pathophysiological understanding of apogeotropic PSC-BPPV has evolved considerably over the past decades. Agus et al. first described an atypical positional downbeating nystagmus with apogeotropic torsional components during Dix–Hallpike testing and attributed it to otoconial debris located within the non-ampullary arm of the posterior canal, generating inhibitory ampullipetal endolymphatic flow [5]. This interpretation was subsequently formalized by Vannucchi et al., who defined the apogeotropic variant of PSC-BPPV [6].

Califano et al. later proposed a classification comprising two subtypes: a non-ampullary arm variant and a periampullary variant [7]. Both configurations generate inhibitory ampullipetal endolymphatic flow and produce a downbeating apogeotropic torsional nystagmus. However, the periampullary subtype may exhibit abrupt conversion to a typical geotropic pattern due to rapid migration of otoconia into the ampullary arm, potentially triggered by positional maneuvers or mastoid vibration.

**Figure 1:**
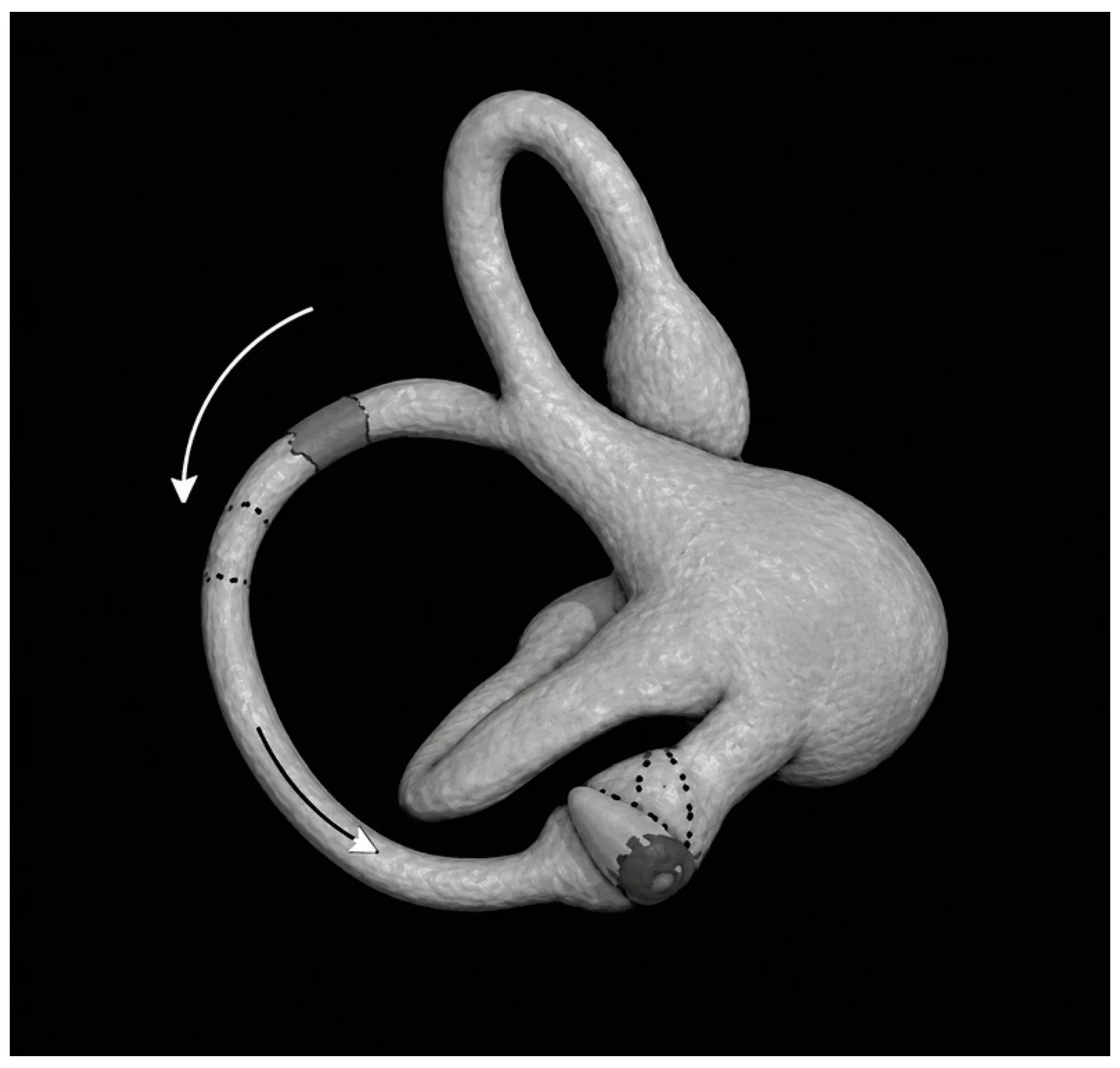
Movement of otoconia during the diagnostic maneuver in apogeotropic posterior semicircular canal BPPV with otoconia located in the non-ampullary arm. Reprinted from [1,7]. The arrow indicate the direction of endolymphatic flow during otoconial movement.

**Figure 2:**
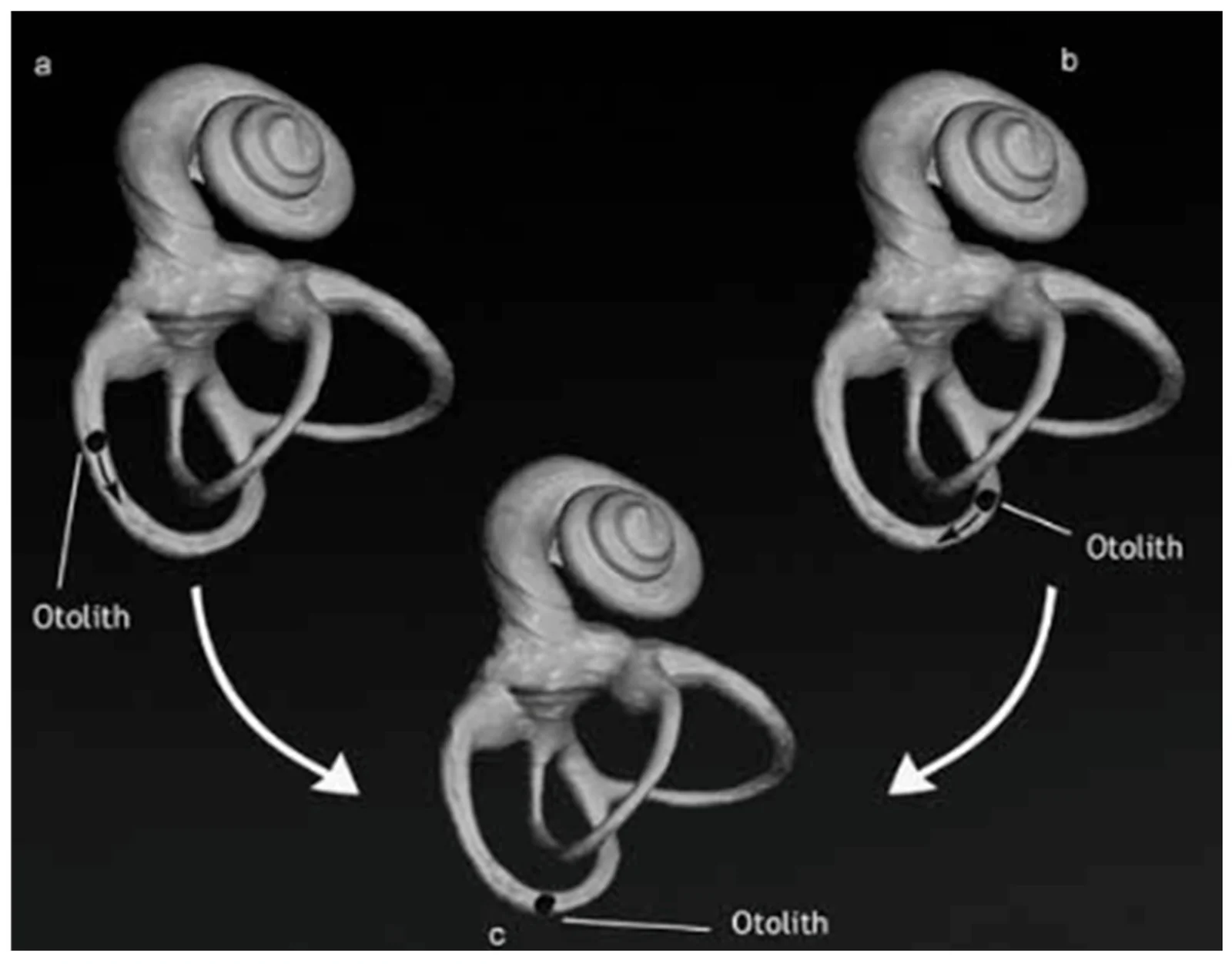
Left posterior canal BPPV: typical versus apogeotropic forms. During the Dix–Hallpike maneuver, otoconia occupy the same position of the canal in both variants. Reprinted from [1,7]. (**a**): otoconial debris in ampullary arm in typical PSC-BPPV. (**b**): otoconial debris in non-ampullary arm in apogeotropic PSC-BPPV. (**c**): otoconial debris position during Dix-Hallpike maneuver.

Geometric models have demonstrated that the final position of the otoconial debris at the end of the Dix–Hallpike maneuver is equivalent in both the geotropic and apogeotropic variants, supporting the use of standard canalith repositioning procedures, including the Epley, Semont, and Quick Liberatory Rotation maneuvers, even in apogeotropic forms [1] (Figure 1 and Figure 2).

Skull vibration applied at 100 Hz over the mastoid process is a well-established bedside test for detecting vestibular asymmetry, typically eliciting a horizontal nystagmus beating toward the healthy side [8]. However, its potential role in influencing otoconial dynamics in BPPV remains poorly understood. In the original description of the canalith repositioning procedure, Epley routinely used vibration as an adjunct to treatment [9]. Subsequently, Faralli et al. proposed mastoid vibration both as a means of converting apogeotropic lateral canal BPPV into the geotropic variant [10] and as a treatment option for an atypical case of posterior canal BPPV [11].

Based on these considerations, the aim of the present study was to investigate whether mastoid vibration facilitates conversion of apogeotropic PSC-BPPV into the typical geotropic form and whether this conversion may improve differential diagnosis from anterior canal BPPV. We also evaluated its potential impact on the number of repositioning maneuvers required to achieve complete symptom resolution.

## 2. Materials and Methods

In this retrospective study, we evaluated the effects of mastoid 100 Hzvibration-induced nystagmus geotropization in ten patients with clinical findings consistent with apogeotropic posterior semicircular canal benign paroxysmal positional vertigo (PSC-BPPV). Six patients were evaluated and treated at the Department of Audiology and Phoniatrics, AORN San Pio Hospital, Benevento, Italy, and four patients were evaluated and treated at the Department of Otolaryngology, IRCCS San Raffaele Scientific Institute, San Raffaele Hospital, Milan, Italy, between September 2024 and April 2026.

To assess the potential efficacy of mastoid vibration in facilitating disease resolution, clinical outcomes were compared with those of a control group consisting of ten patients with apogeotropic PSC-BPPV and comparable clinical characteristics. These patients were selected from individuals treated at the same institutions during the preceding three years. The control group underwent standard treatment with the Demi-Semont liberatory maneuver [12] without adjunctive mastoid vibration.

A second control group consisting of ten patients with typical PSC-BPPV was included to compare the number of repositioning maneuvers required to achieve complete symptom resolution between patients with apogeotropic PSC-BPPV treated with adjunctive mastoid vibration and those with the typical form of PSC-BPPV.

The primary outcome was the proportion of patients demonstrating nystagmus geotropization following mastoid vibration. The secondary outcome was the number of repositioning maneuvers required to achieve complete clinical resolution.

All patients underwent a standardized neuro-otological bedside examination. In patients with a clinical history suggestive of BPPV, ocular motility was assessed first, followed by evaluation of otoconial displacement within the semicircular canals using the Dix–Hallpike and Pagnini–McClure maneuvers [13,14]. The Head-Shaking Test and mastoid vibration test were intentionally not performed before diagnostic positioning or therapeutic repositioning maneuvers in order to avoid inadvertent displacement of otoconial debris within the semicircular canal system prior to completion of the diagnostic assessment.

Patients with a confirmed diagnosis of BPPV underwent follow-up evaluation within five to seven days to assess symptom resolution. In cases of persistent symptoms, additional repositioning maneuvers were performed and follow-up evaluations were repeated at the same interval until complete symptom resolution was achieved. Mastoid vibration was applied over the affected mastoid in all the patients during diagnostic maneuvers (in Dix–Hallpike position in nine patients and in Pagnini–McClure position in one patient with concomitant LSC-BPPV and apogeotropic PSC-BPPV). Once geotropization was achieved, the appropriate liberatory maneuver was immediately performed from the diagnostic position. Following geotropization, patients were treated with either the Epley maneuver [9] or the Quick Liberatory Rotation maneuver [15], according to the treating physician’s clinical judgment. In cases in which geotropization was not achieved, mastoid vibration could be repeated during subsequent therapeutic sessions according to the treating physician’s clinical judgment. The duration of the application of vibration was not predefined and was adapted to the individual clinical response. The duration of vibration was not standardized and depended on patient tolerance and the achievement of geotropization. In some patients conversion occurred within a few seconds, whereas in others several minutes of stimulation were required.

Statistical analyses were performed using Jamovi software (version 2.6.45.0). Data normality was assessed using the Shapiro–Wilk test. Because the distribution of the data was non-normal, between-group comparisons were conducted using the Mann–Whitney U test. Statistical significance was defined as a two-tailed *p*-value < 0.05.

This retrospective observational study was conducted in accordance with the ethical principles of the Declaration of Helsinki. Formal approval by an Institutional Review Board or Research Ethics Committee was not required because the study involved only the retrospective analysis of anonymized clinical data collected during routine clinical practice. No prospective interventions or additional diagnostic procedures were performed for research purposes, and all patients were managed according to established international guidelines.

## 3. Results

Among the ten patients with apogeotropic PSC-BPPV, six (60%) were male and four (40%) were female, resulting in a male-to-female ratio of 1.5:1. The mean age was 53.6 ± 15.3 years (range: 30–79 years).

Regarding medical history, hypertension was present in five patients (50%), hypercholesterolemia requiring pharmacological treatment in six (60%), rheumatoid arthritis in one (10%), cerebellar arteriovenous malformation in one (10%), and type 2 diabetes mellitus in one (10%). Multiple comorbidities were present in several patients, whereas four patients (40%) had no relevant medical history.

A previous history of vestibular disorders was documented in four patients (40%). In the remaining six patients (60%), the current episode represented the first manifestation of vertigo. Among patients with a previous vestibular history, all had experienced prior episodes of PSC-BPPV, although none had reported recurrence within the preceding three months. One 67-year-old patient with rheumatoid arthritis had experienced three previous episodes involving uncommon PSC-BPPV variants, including the Scocco variant [16] and a prior episode of apogeotropic PSC-BPPV.

The affected side was equally distributed, with right-sided involvement in five patients (50%) and left-sided involvement in five patients (50%).

The diagnosis of apogeotropic PSC-BPPV was established in all patients based on the Dix–Hallpike maneuver, which elicited a positional downbeating torsional nystagmus with an apogeotropic pattern in all the patients.

Ipsilateral 100 Hz mastoid vibration was applied during the initial assessment in nine of the ten patients. In the remaining patient, severe nausea precluded adequate mastoid vibration at the initial visit, and mastoid vibration was therefore performed during the follow-up evaluation. In this patient, a Demi-Semont maneuver directed toward the affected side was performed, followed by home-based forced prolonged positioning according to Vannucchi. At follow-up, persistent apogeotropic nystagmus was still present. Mastoid vibration was then applied but failed to induce geotropization after three stimulation cycles of approximately 40 s each. The patient subsequently underwent an Epley maneuver; however, complete symptom resolution was achieved only after an additional Bascule maneuver [17]. This outcome was not considered attributable to mastoid vibration. Once geotropization was achieved, the appropriate liberatory maneuver was immediately performed from the diagnostic position.

Immediate conversion to the geotropic pattern was observed in six of the nine patients (66.7%). The mean duration of vibration required to induce conversion was 70.6 ± 44.8 s (range, 20–160 s). Of these six patients, three exhibited abrupt conversion to the typical geotropic form of PSC-BPPV during vibration, whereas the remaining three demonstrated conversion during repeat Dix–Hallpike testing performed immediately thereafter.

One patient in the active group presented with concomitant apogeotropic lateral canal BPPV. Importantly, this patient showed clear geotropization of the posterior canal following mastoid, allowing the subsequent performance of the Epley liberatory maneuver for posterior canal BPPV. Forced prolonged positioning according to Vannucchi [18] was additionally prescribed specifically for the concomitant lateral canal involvement and did not replace or interfere with the treatment of the posterior canal. At the seven-day follow-up visit, complete resolution of both conditions was documented. Therefore, this case was included in the analysis, as the therapeutic procedure performed for the posterior canal was consistent with that applied to the other patients in the study. Accordingly, the presence of concomitant lateral canal BPPV did not interfere with the assessment of mastoid-vibration-induced geotropization of the posterior canal. In the patients where geotropization was obtained through mastoid vibration, the mean number of liberatory maneuvers to achieve symptom resolution was 1.7 ± 0.49.

The remaining two non-responders required two and four liberatory maneuvers (Epley or Quick Liberatory Rotation maneuvers), respectively, and subsequently achieved complete symptom resolution. The mean number of maneuvers required in this subgroup was 3.0 ± 1.41.

In patients in whom geotropization of the nystagmus with conversion to typical PSC-BPPV was not achieved, no upbeating torsional nystagmus was observed upon returning to the seated position.

Overall, mastoid vibration induced geotropization in seven of the ten patients (70%): six showed immediate conversion during the initial evaluation, whereas one demonstrated delayed conversion at follow-up. The remaining three non-responders achieved complete symptom resolution only after additional repositioning maneuvers.

The first control group consisted of ten patients with apogeotropic PSC-BPPV treated without mastoid vibration. Three patients (30%) were male and seven (70%) were female. The mean age was 57.1 years (range, 44–93 years), and relevant comorbidities were present in seven patients (70%). The mean number of liberatory maneuvers required to achieve complete symptom resolution was 2.9 ± 0.74.

Considering all the study sample (responders and non-responders), the mean number of liberatory maneuvers required to achieve complete symptom resolution was 2.1 ± 0.87.

Compared with the study sample, patients in the first control group required a significantly higher number of liberatory maneuvers (Mann–Whitney U test, *p* = 0.036).

The second control group consisted of ten patients with typical PSC-BPPV. Four patients (40%) were male and six (60%) were female, with a mean age of 55.9 years (range, 36–85 years). Relevant comorbidities were present in six patients (60%). The mean number of liberatory maneuvers required for symptom resolution was 2.1 ± 0.52. No statistically significant difference was observed between this group and the study sample (Mann–Whitney U test, *p* = 0.82).

## 4. Discussion

The present study suggests that ipsilateral 100 Hz mastoid vibration may represent a useful adjunctive tool in the diagnostic and therapeutic management of apogeotropic posterior semicircular canal benign paroxysmal positional vertigo (PSC-BPPV). In our cohort, mastoid vibration was associated with conversion to a typical geotropic posterior canal pattern in 70% of cases, thereby providing additional diagnostic information and supporting subsequent therapeutic decision-making.

Apogeotropic PSC-BPPV remains one of the most challenging variants of BPPV because of its close clinical resemblance to contralateral anterior canal BPPV. Both disorders may present with positional downbeating torsional nystagmus during the Dix–Hallpike maneuver, making accurate localisation of the affected canal difficult in routine clinical practice. In this setting, conversion to a typical geotropic posterior canal pattern may provide a valuable diagnostic clue supporting posterior canal involvement.

In the present series, mastoid vibration was associated with conversion either immediately during stimulation or during subsequent positional testing. These findings suggest that vibration may facilitate displacement of otoconial debris within the posterior canal, thereby promoting conversion to the typical form of PSC-BPPV. Furthermore, patients in whom conversion was achieved required fewer repositioning maneuvers than the historical control group with apogeotropic PSC-BPPV treated without mastoid vibration. Given the small sample size, this observation should be interpreted with caution. Although it does not establish a causal relationship, it suggests a potential therapeutic benefit that warrants further investigation.

The observed findings are consistent with current pathophysiological models of apogeotropic PSC-BPPV. Agus et al. first proposed that this variant results from otoconial debris located within the non-ampullary arm of the posterior canal, producing inhibitory ampullipetal endolymphatic flow during diagnostic positioning [5]. This concept was subsequently refined by Vannucchi et al. [6] and further expanded by Califano et al. [7], who proposed the existence of two distinct subtypes: a non-ampullary arm variant and a periampullary variant.

According to this model, abrupt conversion to a geotropic pattern following mastoid vibration may indicate migration of debris from the periampullary region into the ampullary arm of the canal. Such displacement is expected to generate an ampullofugal excitatory endolymphatic flow, resulting in reversal of the nystagmus pattern. This mechanism may explain the immediate conversion observed in several patients in our series. Conversely, delayed conversion during subsequent Dix–Hallpike testing may reflect progressive mobilisation of otoconia induced by vibration.

Previous authors have proposed several approaches to facilitate the conversion of apogeotropic PSC-BPPV into its typical geotropic form. Califano et al. described spontaneous or maneuver-induced conversion during diagnostic positioning [1,7], whereas Epley originally incorporated vibration into his canalith repositioning procedure [9]. Faralli et al. subsequently reported successful use of mastoid vibration both in apogeotropic lateral canal BPPV and in a resistant case of posterior canal BPPV [10,11]. The present findings provide additional clinical evidence consistent with a possible role for vibration in modifying otoconial dynamics.

From a diagnostic perspective, conversion to a typical geotropic posterior canal pattern may be particularly relevant when differentiating apogeotropic PSC-BPPV from anterior canal BPPV. Although subtle differences in torsional and vertical nystagmus components have been described, these features are not always sufficiently reliable in clinical practice. Demonstration of conversion to the typical posterior canal pattern following mastoid vibration strongly supports the diagnosis of apogeotropic PSC-BPPV and may therefore increase diagnostic confidence. Moreover, apogeotropic PSC-BPPV is thought to be more frequent than ASC-BPPV [19,20]. Furthermore, in accordance with the classification proposed by Califano et al. [1], conversion to the typical form of PSC-BPPV also supports the differential diagnosis from central causes, which are those that may raise the greatest concern for the clinician.

The observed reduction in the number of repositioning maneuvers required to achieve symptom resolution should be interpreted cautiously. Although patients who responded to vibration appeared to improve more rapidly than historical controls, the study was not designed to definitively assess treatment efficacy. Nevertheless, the findings raise the possibility that vibration-assisted mobilisation of otoconia may facilitate subsequent repositioning maneuvers and may thereby shorten the therapeutic course.

Finally, positional downbeating nystagmus should always prompt consideration of central vestibular disorders. However, when conversion to a typical posterior canal pattern is observed, a peripheral vestibular origin becomes substantially more likely. In agreement with Helminski et al. [21], our findings support the concept that positional downbeating nystagmus in the absence of accompanying neurological signs should prompt careful evaluation for uncommon variants of BPPV before central causes are assumed. To this end, we consider conversion to either the typical posterior semicircular canal variant or the geotropic lateral semicircular canal variant to be the diagnostic criterion confirming a peripheral vestibular disorder while excluding concomitant central pathology. This finding fulfills the criteria for the “Definite” grade according to the Califano classification [1,7] (Table 1).

Taken together, these findings suggest that mastoid vibration may represent a simple bedside adjunct capable of increasing diagnostic confidence and facilitating therapeutic management in selected patients with apogeotropic PSC-BPPV. Larger prospective studies are warranted to confirm these preliminary observations.

### Study Limitations

This study has several limitations that should be acknowledged.

First, its retrospective design inherently limits the level of evidence and introduces the potential for selection and observational bias. Consequently, causal inferences regarding the effects of mastoid vibration cannot be established.

Second, the sample size was small, reflecting the rarity of apogeotropic PSC-BPPV in clinical practice. Although this limitation reduces the statistical power of the study, the homogeneity of the diagnostic criteria and the use of standardized diagnostic and therapeutic protocols strengthen the internal consistency of the findings.

Third, the comparison group consisted of historical controls rather than randomly assigned patients. Although baseline demographic and clinical characteristics were broadly comparable between groups, residual confounding cannot be excluded.

Fourth, the proposed pathophysiological interpretation of mastoid-vibration-associated conversion remains speculative and is based primarily on currently accepted theoretical models of apogeotropic PSC-BPPV.

Furthermore, the statistical robustness of the observed differences is limited by the small sample size, particularly in subgroup analyses. Therefore, the findings should be considered exploratory and hypothesis-generating rather than definitive.

Larger cohorts would be desirable to validate these preliminary observations. However, the low prevalence of apogeotropic PSC-BPPV makes patient recruitment challenging, even in referral centers. Adequately powered prospective studies will likely require multicenter collaboration and prolonged recruitment periods.

Despite these limitations, the present study provides preliminary clinical evidence that mastoid vibration may facilitate geotropization in selected patients with apogeotropic PSC-BPPV and may provide additional diagnostic information to support clinical management. These findings should be regarded as hypothesis-generating and warrant confirmation in larger prospective studies.

## 5. Conclusions

Ipsilateral 100 Hz mastoid vibration appears to be a useful adjunctive tool in the management of apogeotropic posterior semicircular canal benign paroxysmal positional vertigo (PSC-BPPV), with potential diagnostic and therapeutic implications.

In the present series, mastoid vibration was associated with conversion to a typical geotropic posterior canal pattern in 70% of patients. This finding may provide clinically useful diagnostic information and assist subsequent therapeutic decision-making. In particular, demonstration of a typical geotropic posterior canal response following mastoid vibration may facilitate differentiation between apogeotropic PSC-BPPV and contralateral anterior canal BPPV, thereby increasing diagnostic confidence in patients presenting with positional downbeating torsional nystagmus.

Patients who demonstrated conversion to a geotropic pattern also required fewer repositioning maneuvers than historical controls with apogeotropic PSC-BPPV, suggesting a potential therapeutic benefit. However, this finding should be interpreted cautiously given the retrospective design, the limited sample size, and the use of non-randomized control groups.

Overall, these preliminary findings support the hypothesis that mastoid vibration may facilitate otoconial mobilization and enhance the diagnostic and therapeutic management of apogeotropic PSC-BPPV. Given the limited sample size, the present findings should be interpreted primarily as descriptive and hypothesis-generating rather than as evidence of comparative therapeutic efficacy. If confirmed in larger prospective studies, mastoid vibration may represent a simple, inexpensive, and readily available bedside adjunct capable of improving both diagnostic accuracy and treatment efficiency in patients with apogeotropic PSC-BPPV.

## Figures and Tables

**Table 1:** Classification proposed by Califano et al. for Apogeotropic PSC-BPPV [1]. APC BPPV: apogeotropic posterior benign paroxysmal positional vertigo; MRI: magnetic resonance imaging.

(a) Definite APC BPPV
Presence of torsional apogeotropic nystagmus, evoked through the Dix–Hallpike test and sometimes through the straight head hanging positioning. Presence of a vertical downbeating component in the same positioning tests. Canalar conversion in typical posterior canal BPPV during or immediately after (no more than two days) the therapeutic maneuver, or suddenly during the prolonged maintenance of one of the head hanging positions (straight head hanging; “deep” Dix–Hallpike position) or during the maintenance of leaning position in sitting position.
(b) Probable APC BPPV
As reported in a, but with a direct resolution of disease without canalar conversion in typical posterior canal BPPV.
(c) Possible APC BPPV
Persistence of symptoms after 5 cycles of therapeutic maneuvers, MRI does not show any alterations of the central nervous system as a presumed cause of the nystagmus, or patient lost to follow-up before the resolution of the disease.

## Data Availability

The original contributions presented in this study are included in the article. Further inquiries can be directed to the corresponding author.
